# Supplementary material for: Complex‐centric proteome profiling by SEC‐SWATH‐MS
Source: Mol Syst Biol. 2019 Jan 14;15(1):e8438. doi: 10.15252/msb.20188438 (PMC6346213; doi:10.15252/msb.20188438)
Supplement: Supplementary file 8 — Dataset EV7 [file MSB-15-e8438-s008.zip › feature_plots_string/Q99547.pdf]

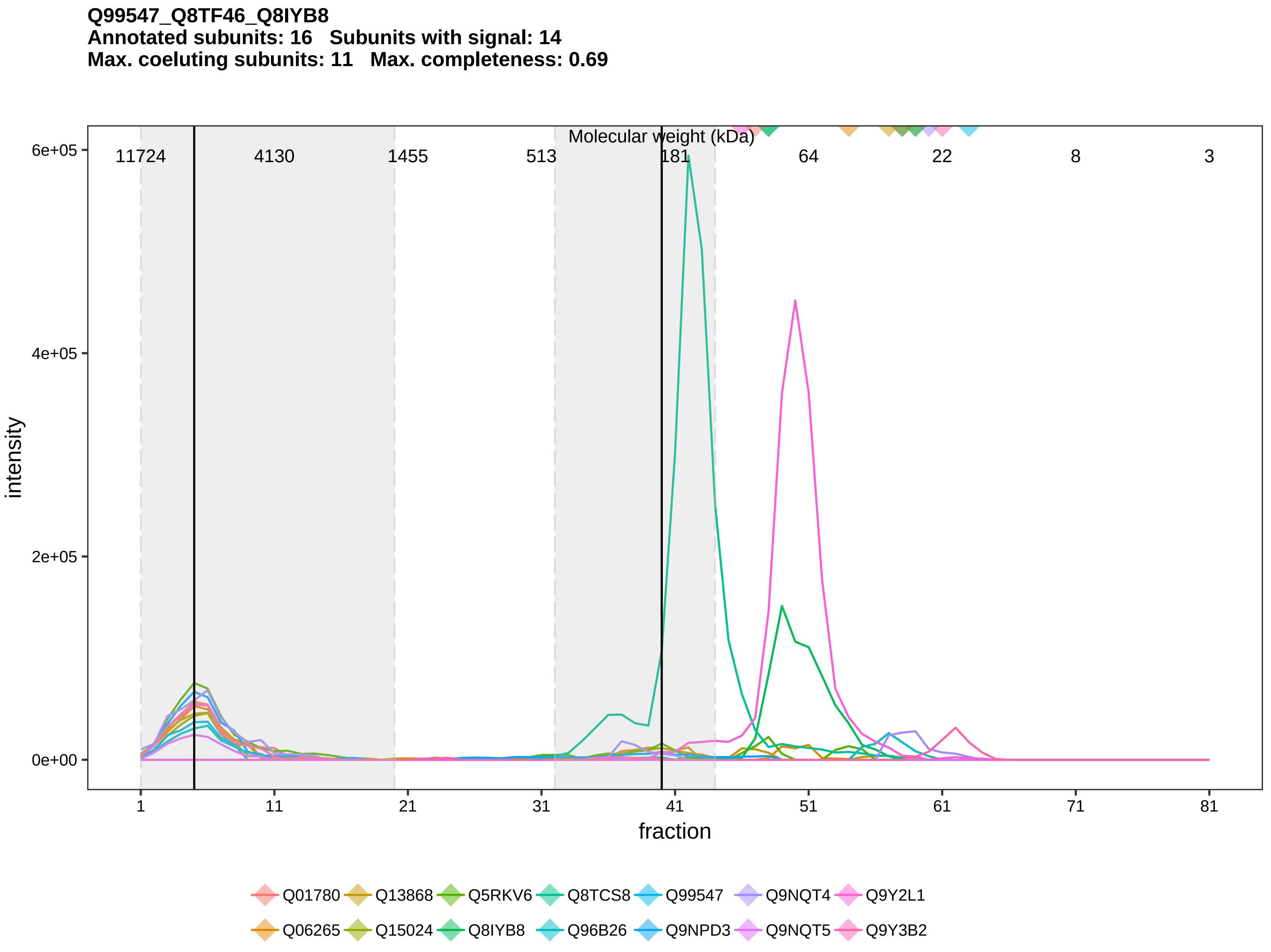
Q99547_Q8TF46_Q8IYB8
Annotated subunits: 16 Subunits with signal: 14
Max. coeluting subunits: 11 Max. completeness: 0.69
Molecular weight (kDa)
11724
4130
1455
513
181
64
22
8
3
6e+05
4e+05
2e+05
0e+00
intensity
1
11
21
31
41
51
61
71
81
fraction
Q01780
Q13868
Q5RKV6
Q8TCS8
Q99547
Q9NQT4
Q9Y2L1
Q06265
Q15024
Q8IYB8
Q96B26
Q9NPD3
Q9NQT5
Q9Y3B2
